# Binding of S100A6 to actin and the actin–tropomyosin complex

**DOI:** 10.1038/s41598-020-69752-y

**Published:** 2020-07-30

**Authors:** Ewelina Jurewicz, Katarzyna Robaszkiewicz, Joanna Moraczewska, Anna Filipek

**Affiliations:** 1 Nencki Institute of Experimental Biology, Polish Academy of Sciences, 3 Pasteur Street, 02-093 Warsaw, Poland; 2 Department of Biological Sciences, Kazimierz Wielki University, 12 Poniatowskiego Street, 85-671 Bydgoszcz, Poland

**Keywords:** Biochemistry, Cell biology

## Abstract

S100A6 is a low molecular weight Ca^2+^-binding protein belonging to the S100 family. Many reports indicate that in the cell S100A6 has an influence on the organization of actin filaments, but so far no direct interaction between S100A6 and actin has been shown. In the present study we investigated binding of S100A6 to actin and the actin–tropomyosin complex. The analyses were performed on G- and F-actin and two tropomyosin isoforms—Tpm1.6 and Tpm1.8. Using purified proteins and a variety of biochemical approaches we have shown that, in a Ca^2+^-bound form, S100A6 directly interacts with G- and F-actin and with tropomyosin, preferentially with isoform Tpm1.8. S100A6 and tropomyosin bind to the same population of filaments and the presence of tropomyosin on the microfilament facilitates the binding of S100A6. By applying proximity ligation assay we have found that in NIH3T3 fibroblasts S100A6 forms complexes both with actin and with tropomyosin. These results indicate that S100A6, through direct interactions with actin and tropomyosin, might regulate the organization and functional properties of microfilaments.

## Introduction

S100A6 is a Ca^2+^-binding protein belonging to the S100 protein family^1^. S100A6 gene expression is cell specific and, in vitro, can be induced by multiple agents, among others, by stress factors^2^*.* The protein is present in different mammalian tissues and cells. Particularly, its high expression is found in mouse stomach and chicken gizzard^3,4^. As to cell distribution, the protein is expressed mainly in fibroblasts and epithelial cells^5^. Many reports which appeared up to now have shown that S100A6 plays multiple cellular functions^6^. For instance, it has been demonstrated, that S100A6 overexpression leads to higher cell proliferation rate and sensitizes cells to apoptosis^7,8^. On the other hand, diminished level of S100A6 not only inhibits proliferation but also alters cell morphology^9^*.* In particular, S100A6 deficiency in fibroblasts has led to changes in the shape, adhesion and motile properties of these cells, probably due to rearrangements in actin stress fiber architecture and/or reorganization of microfilaments associated with tropomyosin^9,10^.

Tropomyosins are actin regulatory proteins, which polymerize on both sides of the filament, stabilize the filament and control interactions of actin with other binding partners. In mammalian cells about forty tissue-specific and constitutive isoforms of tropomyosin are expressed^11,12^. The isoforms segregate to different cell compartments and diversify microfilaments’ properties such as stability, dynamic polymerization and depolymerization, interactions with myosin motors and other actin-binding proteins^13,14^*.* S100A6 was shown to interact in vitro with smooth muscle tropomyosin^15^, therefore it is possible that changes in microfilament architecture observed in S100A6-deficient NIH3T3 fibroblasts were coordinated by tropomyosins. Although many reports have indicated that S100A6 has an influence on the rearrangement of actin filaments, so far, no direct interaction of S100A6 with actin was clearly demonstrated. It was also not known whether tropomyosin affects S100A6 interaction with microfilaments.

The goal of this work was to check whether S100A6 directly interacts with actin, tropomyosin and the actin–tropomyosin complex. Since, in the cell, actin is in a dynamic equilibrium between a monomeric (G-actin) and filamentous (F-actin) form, we examined interaction of S100A6 with both forms of actin, and with F-actin covered by various tropomyosin isoforms. We selected two non-muscle tropomyosin isoforms, Tpm1.6 and Tpm1.8, which co-localize with actin filaments in different cell compartments^16^. For such studies we used purified proteins and applied different biochemical approaches. To check whether the interactions also occur in the cell we applied proximity ligation assay (PLA) in NIH3T3 fibroblasts.

## Results

### Analysis of S100A6–actin interaction

**Figure 1:**
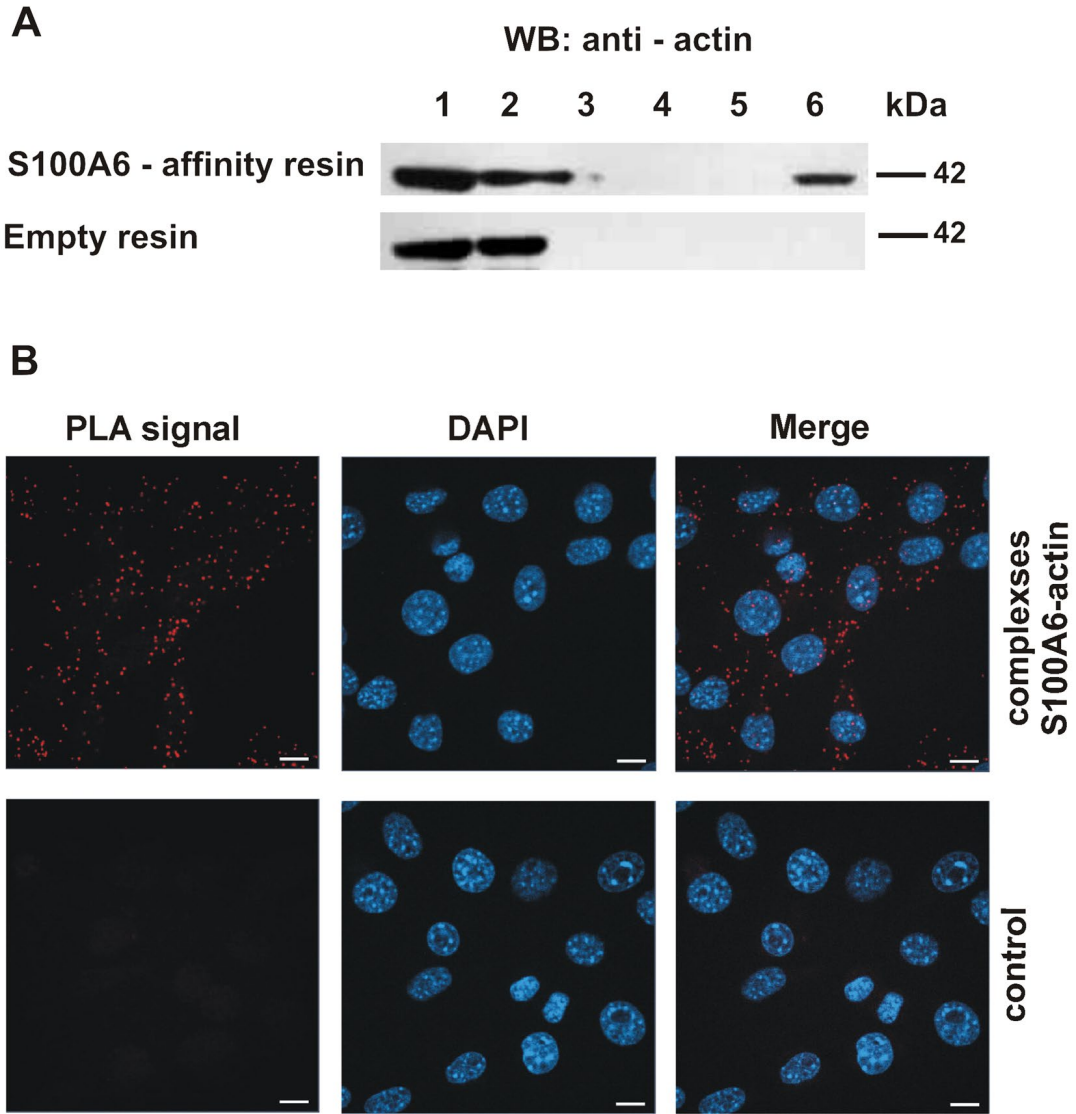
Interaction of S100A6 with actin in NIH3T3 fibroblasts. (**A**) Pull-down assay with the use of protein lysate from NIH3T3 cells and CNBr-Sepharose-S100A6 affinity resin (upper panel) or CNBr-Sepharose-empty resin (lower panel). Lanes: 1—input, 2—unbound fraction, 3—last wash, 4—first wash with 250 mM NaCl, 5—last wash with 250 mM NaCl, 6—elution. Fractions were analyzed by SDS-PAGE (10% gel) followed by immunoblotting developed with anti-actin antibodies. (**B**) Presence of S100A6–actin complexes in NIH3T3 fibroblasts analyzed by PLA assay. Control represents the experiment in which ligase was omitted. Complexes of examined proteins are visualized in red; cell nuclei, stained with DAPI, are in blue. Scale bar is 10 μm.

To check whether S100A6 is capable of binding to actin, a pull-down assay with the use of CNBr-Sepharose-S100A6 affinity resin and a lysate prepared from NIH3T3 fibroblasts, was conducted. Proteins bound to the resin in a Ca^2+^-dependent manner and eluted in buffer containing EGTA were analyzed by immunoblotting using anti-actin antibody. As it can be seen in Fig. 1A, a band corresponding to actin is present in the elution fraction (upper panel, lane 6), which suggests that in NIH3T3 fibroblasts the two proteins interact with each other. To confirm that S100A6 forms complexes with actin in the cell we performed proximity ligation assay (PLA). The results demonstrated the presence of actin–S100A6 complexes in the cytoplasm of NIH3T3 cells (Fig. 1B, upper panel). In control experiments, performed either in the absence of ligase (Fig. 1B, lower panel) or in the absence of primary antibodies (not shown), no signals were detected.

**Figure 2:**
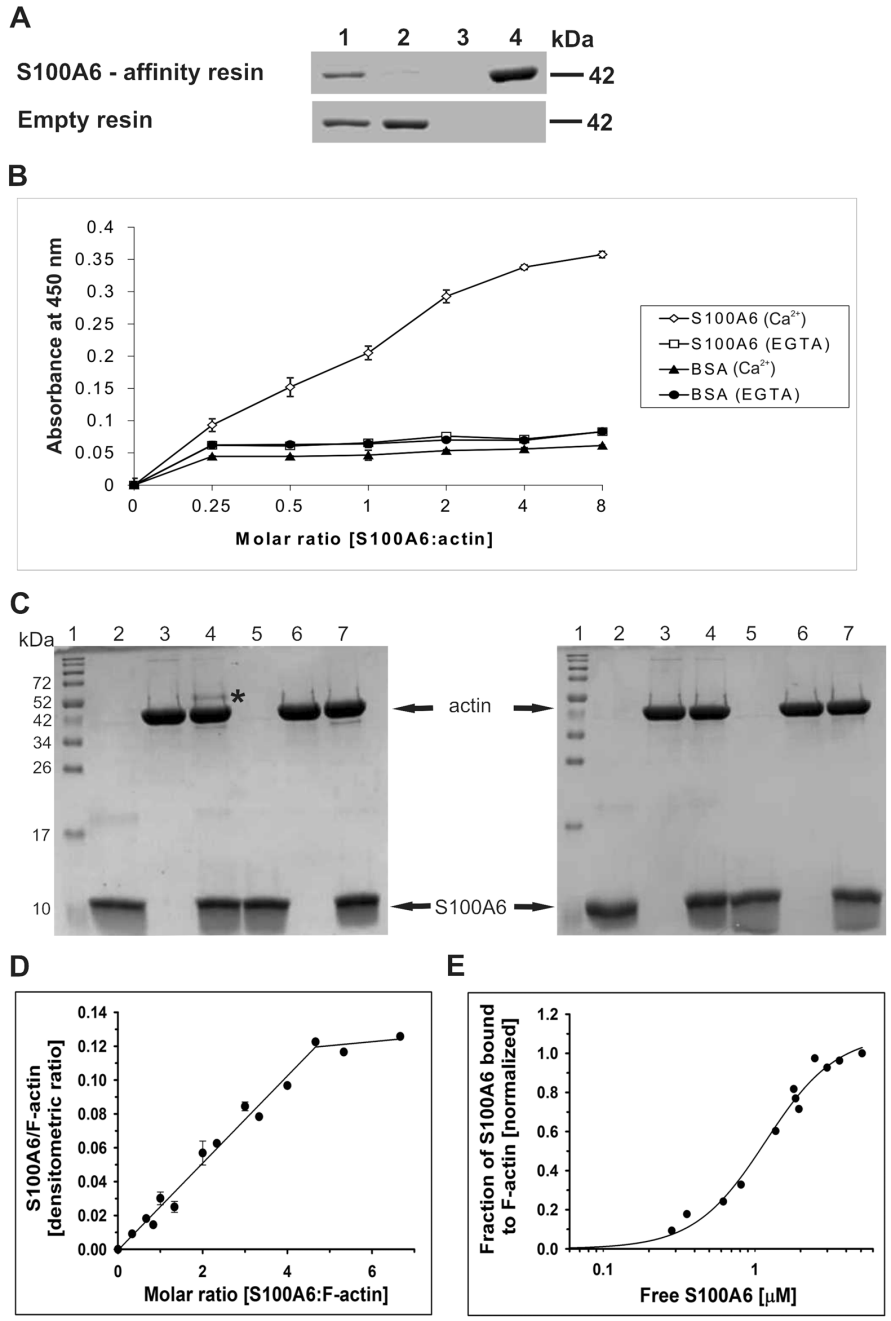
Interaction of S100A6 with purified G-actin. (**A**) Pull-down assay performed with purified G-actin and with CNBr-Sepharose-S100A6 affinity resin (upper panel) or CNBr-Sepharose-empty resin (lower panel). Lanes: 1—input, 2—unbound fraction, 3—last wash, 4—elution. Fractions were analyzed by SDS-PAGE (10% gel) stained with Coomassie brilliant blue R250. (**B**) ELISA assay. The amount of S100A6 bound to G-actin was estimated using anti-S100A6 antibodies. The points represent mean absorbance values ± SD from 3 independent experiments. (**C**) Chemical cross-linking of S100A6 with G-actin analyzed by SDS-PAGE (15% gel) stained with Coomassie brilliant blue R250. Lanes 2 and 5—S100A6 alone, lanes 3 and 6—G-actin alone and lanes 4 and 7—mixture of S100A6 and G-actin. Proteins were incubated with EDC (lanes 2–4) or without this agent (lanes 5–7) in the presence of Ca^2+^ (left panel) or in their absence (right panel). Lanes no. 1 show the position of molecular weight standards (broad range, Bio-Rad). “*” indicates the S100A6–actin cross-linking product. (**D**) Saturation of F-actin with S100A6 analyzed by a co-sedimentation assay. S100A6:actin densitometric ratio drawn as a function S100A6:F-actin molar ratio. The experimental points were fit to linear regression. (**E**) Fractional saturation of F-actin with S100A6 as a function of unbound S100A6. The binding curve was obtained by fitting the experimental points to the Hill equation (Eq. 1). Analyzed data are from 3 independent experiments. Conditions: 3 μM F-actin, 0–20 μM S100A6, 10 mM Tris, pH, 7.5, 50 mM NaCl and 1 mM CaCl_2_.

In the next step we established whether S100A6 interacts with G-actin and/or with F-actin, and if the binding is direct and Ca^2+^-dependent. For that, purified G-actin was applied to the CNBr-Sepharose-S100A6 affinity resin in a buffer containing Ca^2+^. Proteins bound in the presence of Ca^2+^ were then eluted in a buffer containing EGTA. As it is shown in Fig. 2A, a band corresponding to G-actin was present in the elution fraction from the CNBr-Sepharose-S100A6 affinity resin (upper panel, lane 4) but not in the corresponding fraction from the control CNBr-Sepharose-empty resin (lower panel, lane 4).

Interaction between S100A6 and G-actin was additionally tested by an ELISA assay, which was done with the use of purified proteins. As illustrated in Fig. 2B, much higher absorbance was registered when S100A6 was added to the wells coated with G-actin than to those coated with BSA, which suggests that the S100A6-actin interaction is specific. Also, a higher absorbance was registered when the proteins were incubated in a buffer containing Ca^2+^.

The interaction between S100A6 and G-actin was also examined by chemical cross-linking of both proteins using EDC in the presence or in the absence of Ca^2+^. As shown in Fig. 2C, a 52-kDa cross-linking product was formed in the presence of Ca^2+^ (left panel, lane 4, asterisk), but not in the absence of Ca^2+^ (right panel, lane 4) or in the control sample without EDC (left panel, lane 7). Thus, the results from pull-down assay, ELISA and cross-linking indicate that the S100A6–G-actin interaction is direct and Ca^2+^-dependent.

Then, we analyzed binding of S100A6 to filamentous F-actin. For that an in vitro co-sedimentation assay with the use of purified proteins was performed. As illustrated in Fig. 2D, S100A6 bound to F-actin, but as much as fivefold molar excess of S100A6 over actin was required to reach saturation. The experimental points were fit to a linear regression showing that half-maximal saturation was obtained at a 2.5 molar ratio of S100A6 to F-actin. In order to determine the apparent binding constant (K_app_), the fractional saturation of F-actin with S100A6 was drawn as a function of free S100A6 (Fig. 2E). Fitting the experimental points to Eq. 1 allowed us to compute the K_app_ and the Hill cooperativity coefficient, which is a measure of cooperation between S100A6 molecules bound to actin (Table 1). The obtained parameters indicate that binding of S100A6 to F-actin is weak, but cooperative.

**Table 1:** Effects of tropomyosin isoforms on the parameters of S100A6 binding to F-actin.

	K_app_ [× 10^6^ M^−1^]	α^H^
F-actin	0.9 ± 0.1	1.9 ± 0.3
F-actin + Tpm1.6	1.3 ± 0.2	1.2 ± 0.2
F-actin + Tpm1.8	1.6 ± 0.2*	1.5 ± 0.1

**Figure 3:**
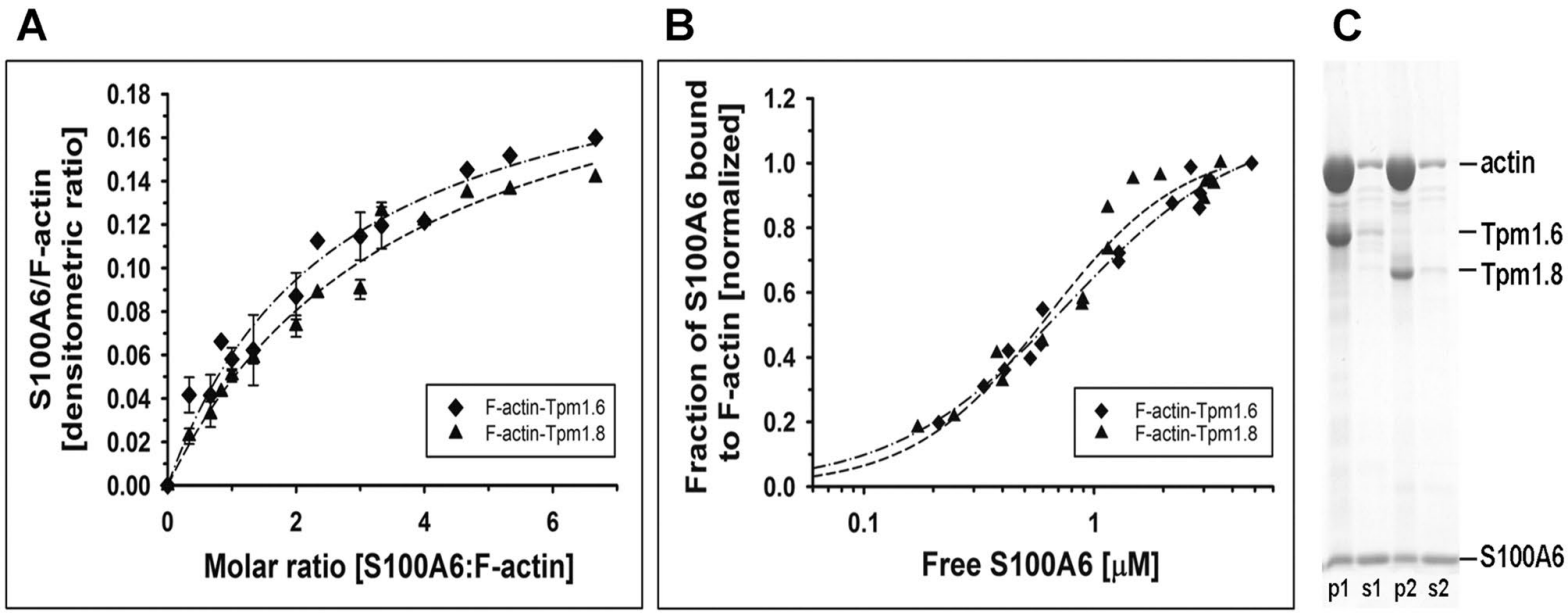
Co-sedimentation of S100A6 with F-actin–tropomyosin complex. (**A**) Saturation of the F-actin–tropomyosin complex with S100A6 as a function S100A6:F-actin molar ratio. The experimental points were fit to hyperbolic equation. (**B**) Fractional saturation of F-actin with S100A6 shown as a function of unbound S100A6. The experimental points were fit to the Hill equation (Eq. 1). Conditions: 3 μM F-actin, 0.5 µM tropomyosin, 0–20 µM S100A6, 10 mM Tris, pH, 7.5, 50 mM NaCl and 1 mM CaCl_2._ The data are from 3 independent experiments. (**C**) Proteins collected in pellets (p) and supernatants (s) at saturation point, separated in SDS-PAGE (12% gel). 3 μM F-actin saturated with either 0.5 µM Tpm1.6 (p1, s1) or 0.5 µM Tpm1.8 (p2, s2) and 14 µM S100A6.

The parameters were calculated from the binding curves obtained by fitting Eq. 1 to experimental points collected from three independent experiments. Protein binding conditions were as described in the “Materials and methods” section and in the caption to Figs. 2 and 3. *Statistically significant difference in S100A6 binding to F-actin in the absence and presence of tropomyosin (*p* ≤ 0.01).

### Binding of S100A6 to the F-actin–tropomyosin complex

Most of the microfilaments present in various types of cells are associated with tropomyosin^17^, therefore we checked whether S100A6 binds to the F-actin–tropomyosin complex. For this, co-sedimentation of S100A6 with F-actin saturated with tropomyosin isoforms, Tpm1.6 and Tpm1.8, was performed. The saturating concentrations of both tropomyosin isoforms were selected on the basis of previously obtained tropomyosin–actin binding parameters^18^*.* The binding curves illustrated in Fig. 3 show that S100A6 bound to F-actin saturated with each tropomyosin isoform. The experimental points illustrated in Fig. 3A departed from a linear fit, which was characteristic for S100A6 binding to F-actin in the absence of tropomyosin (Fig. 2D), and were best fit to hyperbolic equation. As a result, the concentration of S100A6 required for half maximal saturation was reduced from a 2.5-molar excess of S100A6 over actin in the absence of tropomyosin (Fig. 2D) to a 1.5-molar excess of S100A6 in the presence of tropomyosin (Fig. 3A). The K_app_ was computed by fitting the experimental points illustrated in Fig. 3B to Hill equation (Eq. 1 in “Materials and methods” section). The binding parameters collected in Table 1 showed that both tropomyosin isoforms tended to increase the affinity of S100A6 to F-actin, but the results were statistically significant only in the presence of Tpm1.8. None of the tropomyosin isoforms significantly changed the Hill coefficient (α^H^) of S100A6 binding to F-actin. However, one should note that the experimental points obtained in the presence of Tpm1.8 are scattered, which affects fitting the data and decreases significance of the differences in the binding parameters.

The presence of S100A6 and each tropomyosin isoform on the actin filament at saturation point is illustrated in Fig. 3C. As it can be seen in this figure all three proteins were present in pellets obtained in the co-sedimentation assay, which indicates that S100A6 does not compete with tropomyosin for the binding sites on actin. Based on these results one can conclude that the presence of tropomyosin facilitates interaction of S100A6 with microfilaments.

### Analysis of the S100A6–tropomyosin interaction

**Figure 4:**
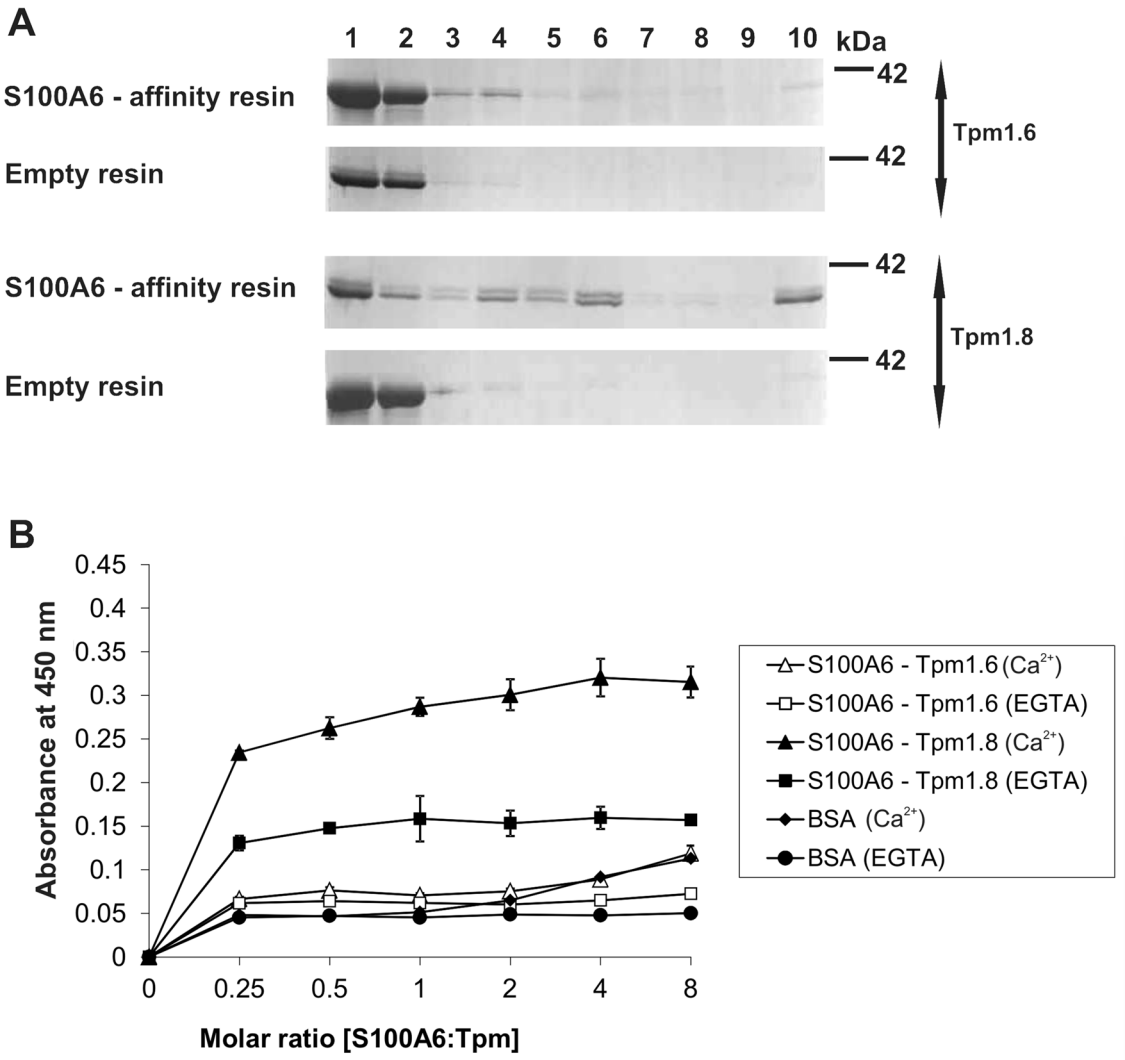
Interaction of S100A6 with tropomyosin isoforms. (**A**) Pull-down assay with the use of purified Tpm1.6 or Tpm1.8 and with CNBr-Sepharose-S100A6 affinity resin (upper panels) or CNBr-Sepharose-empty resin (lower panels). Lanes: 1—input, 2—unbound fraction, 3—last wash, 4—first wash with 150 mM NaCl, 5—last wash with 150 mM NaCl, 6—first wash with 500 mM NaCl, 7—last wash with 500 mM NaCl, 8—first wash with 1 M NaCl, 9—last wash with 1 M NaCl, 10—elution. Fractions were analyzed by SDS-PAGE (10% gel) stained with Commassie brilliant blue R250. (**B**) ELISA assay. The amount of S100A6 bound to Tpm was estimated using anti-S100A6 antibodies. The points represent mean absorbance values ± SD from 3 independent experiments.

To find the reason of the increased affinity of S100A6 to F-actin covered with Tpm1.8, we checked whether S100A6 directly interacts with tropomyosin in vitro and in NIH3T3 fibroblasts. For that, purified Tpm1.6 and Tpm1.8 were applied to CNBr-Sepharose-S100A6 affinity resin in a buffer containing Ca^2+^, followed by elution of bound proteins with a buffer containing EGTA. As shown in Fig. 4A, both tropomyosin isoforms were detected (all panels, lane 1). A weak band corresponding to Tpm1.6 can be seen in the elution fraction from the CNBr-Sepharose-S100A6 affinity resin (1st panel from the top, lane 10). However, the density of the eluted Tpm1.6 band was only a small fraction of the density of Tpm1.6 loaded on the resin (1st panel from the top, lane 1). Binding of Tpm1.8 to the S100A6 affinity resin was much more robust. The density of the Tpm1.8 band eluted from the CNBr-Sepharose-S100A6 affinity resin (3rd panel from the top, lane 10) was about half of the density of the band representing the loaded Tpm1.8 (3rd panel from the top, lane 1). None of the tropomyosin isoforms bound to the CNBr-Sepharose-empty resin (2nd and 4th panels from the top, lanes 10), which showed that binding to the S100A6 coupled to CNBr-Sepharose resin was specific.

To further analyze direct interaction between S100A6 and tropomyosin isoforms, an ELISA assay was performed using purified proteins. The results illustrated in Fig. 4B confirmed that S100A6 binds much stronger to Tpm1.8 and that the binding was Ca^2+^-dependent. In this assay binding of Tpm1.6 to S100A6 was very weak.

**Figure 5:**
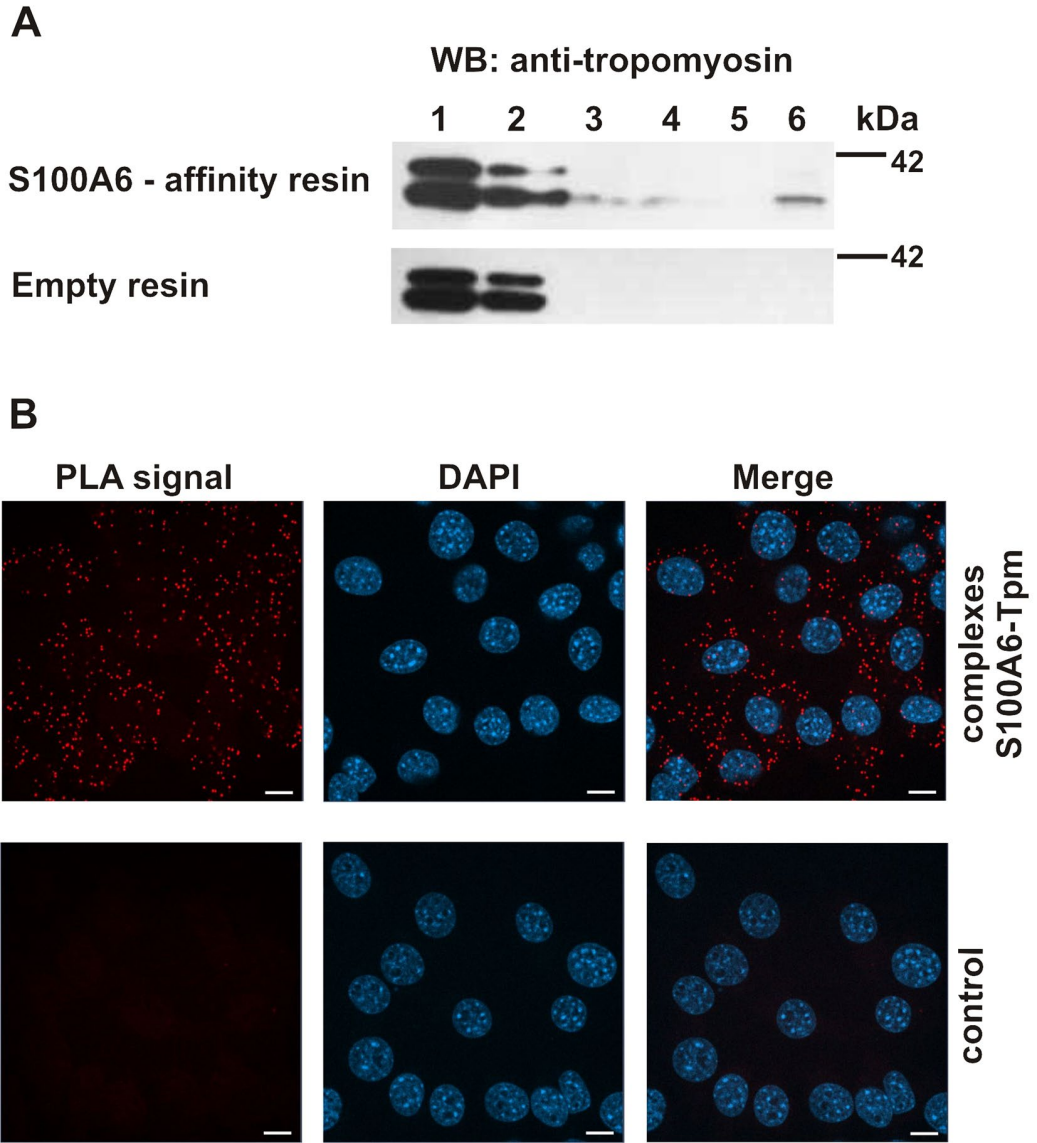
Interaction of S100A6 with tropomyosin in NIH3T3 fibroblasts. (**A**) Pull-down assay with the use of protein lysate from NIH3T3 fibroblasts and with CNBr-Sepharose-S100A6 affinity resin (upper panel) or CNBr-Sepharose-empty resin (lower panel). Lanes: 1—input, 2—unbound fraction, 3—last wash, 4—first wash with 250 mM NaCl, 5—last wash with 250 mM NaCl, 6—elution. Fractions were analyzed by SDS-PAGE (10% gel) followed by immunoblotting developed with mouse monoclonal anti-tropomyosin antibody, Sigma Aldrich, clone TM311. (**B**) Presence of S100A6–Tpm complexes in NIH3T3 fibroblasts analyzed by PLA assay. Control represents the experiment in which ligase was omitted. Complexes of examined proteins are visualized in red; cell nuclei, stained with DAPI, are in blue. Scale bar is 10 μm.

Subsequently, we tested S100A6–tropomyosin interaction in cell lysate obtained from NIH3T3 fibroblasts. For that the protein lysate was applied to the CNBr-Sepharose-S100A6 affinity resin in a buffer containing Ca^2+^. Proteins bound in the presence of Ca^2+^ and eluted in a buffer containing EGTA were then analyzed by SDS-PAGE and immunoblotting using anti-tropomyosin antibodies (Fig. 5A). In the lysate obtained from NIH3T3 fibroblasts the anti-tropomyosin antibody detected two protein bands (lane 1). However, only the lower molecular weight tropomyosin was bound to the CNBr-Sepharose-S100A6 affinity resin in the presence of Ca^2+^ and was eluted in EGTA-containing buffer (upper panel, lane 6). No such band was eluted from the CNBr-Sepharose-empty resin (lower panel, lane 6).

Finally, we checked whether S100A6 can form complexes with tropomyosin in the cell. For that we performed proximity ligation assay (PLA) in NIH3T3 fibroblasts (Fig. 5B). The obtained results indicate that, as in vitro, S100A6 forms complexes with tropomyosin, which are visible as red dots. Similarly as in the case of the S100A6-actin interaction detected by PLA, the S100A6-tropomyosin complexes were evenly distributed in the cytoplasm (upper panel). In control experiments, when ligase was omitted (lower panel) or when cells were incubated without primary antibodies (not shown), no specific signals (red dots) were observed.

## Discussion

S100A6 (formerly called calcyclin) is a Ca^2+^-binding protein belonging to the S100 family^1,6^. At present, this family consists of 25 members which exhibit tissue- and cell- specific distribution. S100 proteins, through interactions with various target proteins, are involved in many cellular functions such as regulation of Ca^2+^ homeostasis, proliferation, differentiation, apoptosis, inflammation, migration/invasion etc. Of note, S100A6 ligands include some components of the cytoskeleton such as tropomyosin, tubulin or CacyBP/SIP^6^*,* which suggests that S100A6 is involved in cytoskeletal organization and dynamics. This possibility is supported by the earlier observation that S100A6 deficiency in fibroblasts led to altered cell morphology, rearrangement of actin stress fibers, increased adhesion and motility of these cells^9,10^. Because cell shape and motility depend on actin filament dynamics, the above mentioned observations suggest that S100A6 is involved in the maintenance of microfilament architecture. However, it was not known whether S100A6 affects actin filaments through signaling pathways, activation of accessory proteins or through direct interactions with actin. Thus, in this work we examined this issue and showed for the first time that S100A6 binds to actin directly and in a Ca^2+^-dependent way. Moreover, our studies revealed that interaction between S100A6 and the actin filament is facilitated by tropomyosin—a regulatory protein, which controls actin interactions with many binding partners.

We have shown that S100A6 forms complexes with actin in NIH3T3 fibroblasts. Pull-down assay with the use of CNBr-Sepharose-S100A6 affinity resin indicated that S100A6 bound to actin in cell lysates while the PLA assay revealed a dotted pattern of the S100A6–actin complexes visible throughout the cytoplasm of NIH3T3 fibroblasts. Fibroblasts are highly motile cells, in which actin filaments undergo dynamic polymerization and depolymerization^19^. Because this process is based on a transition from monomeric G-actin to filamentous F-actin, we checked whether S100A6 binds to G-, F- or both forms of actin. Pull-down assay, zero-length cross-linking and ELISA revealed that S100A6 binds to purified monomeric G-actin. Co-sedimentation assay demonstrated that S100A6 binds also to F-actin. For saturation of the filament, 5-molar excess of S100A6 was required, which indicates weak interactions with the apparent binding constant (K_app_) in the micromolar range, however, at this stage of investigations, the binding stoichiometry is unknown. Our experiments revealed a substantial degree of cooperativity of S100A6 binding to the filament. The cooperativity may arise from direct interactions between S100A6 molecules bound along the actin filament, which is possible in view of the S100A6 ability to form dimers^20^. An alternative explanation is that binding of S100A6 induces conformational changes in actin subunits, which are spread along the filament and facilitate S100A6 binding. Structural studies are necessary to get more information on the mechanism of F-actin-S100A6 interactions.

The experiments with purified, G- and F-actin, proved that the binding did not require any accessory proteins. Also, our data showed that S100A6 binds both to β-actin present in the cell lysate and to purified skeletal muscle α-actin used in in vitro assays. This suggests that interaction of S100A6 with actin is a universal feature of this protein, and does not depend on the actin isoform, which may differ in various cell and tissue types.

Earlier studies demonstrated a connection between S100A6 expression level and the specific architecture of the actin cytoskeleton. In fibroblasts transfected with anti-sense S100A6 retrovirus, microfilaments associated with tropomyosin were arranged diagonally across the cell, which was in contrast to the parallel organization in the control fibroblasts expressing normal level of S100A6^9^*.* Because smooth muscle tropomyosin was shown to interact with S100A6 in vitro^15^, tropomyosin can be considered as a potential regulator of S100A6–actin interaction. Fibroblasts express numerous tropomyosin isoforms which segregate to different cellular compartments^16,21^*.* Tropomyosin isoforms, Tpm1.6 and Tpm1.8, selected for this study were shown to associate with different microfilaments. While Tpm1.6 segregates to contractile and non-contractile stress fibers^22^, Tpm1.8 is recruited to dynamic filaments at the base of ruffling membranes^23^ and de-branched filaments of lamellipodia^24^. Tpm1.6 and Tpm1.8 are products of the *TPM1* gene and are identical in sequence except for the N-terminal segment. In spite of the similarity, these isoforms differ in the regulation of actin interactions with actin-binding proteins such as myosin and cofilin^18,25^*.*

In this work we demonstrated that both tropomyosin isoforms facilitated binding of S100A6 to actin filaments by decreasing S100A6 concentration required for half-maximal saturation. This might be a result of conformational changes in F-actin brought about by binding of tropomyosin. Such possibility finds support in the results of our recent study showing tropomyosin-induced changes at the interface between actin subunits along and across the filament, which affected binding of cofilin-1 to actin^26^. The results of CNBr-Sepharose-S100A6 affinity and ELISA assays performed with the use of purified tropomyosin isoforms demonstrated much stronger interaction of S100A6 with Tpm1.8 than with Tpm1.6. Furthermore, the CNBr-Sepharose-S100A6 affinity resin pulled-down only a low molecular isoform of tropomyosin from the NIH3T3 cell lysate. One has to note that in the cell lysate the mouse monoclonal anti-tropomyosin antibody, clone TM311, detected high and low molecular weight tropomyosin isoforms. There is a high probability that the low molecular weight isoform which was pulled-down from the cell lysate was either Tpm1.8 or Tpm1.9, because Tpm1.8 and Tpm1.9 (an isoform which differs from Tpm1.8 in the sequence of an inner segment) are the only two low molecular weight products of the *TPM1* gene found in non-nervous cells^12,16^. This suggests that for specific interactions with tropomyosin S100A6 prefers the N-terminal segment encoded by the alternative exon 1b, which is present in low molecular weight tropomyosin isoforms.

The observation that S100A6 forms complexes with actin and tropomyosin in NIH3T3 fibroblasts strongly supports the idea that interactions between these proteins regulate cytoskeletal organization in vivo*.* As mentioned above, in cells, Tpm1.8 associates mostly with highly dynamic microfilaments^23,24^. Because in this work we demonstrated that Tpm1.8 directly binds to S100A6, we propose that it recruits S100A6 to the filaments which undergo dynamic polymerization and depolymerization required for cell proliferation. As depletion of S100A6 in NIH3T3 fibroblasts resulted in profound stress fibers formation and stronger adhesion^10^, one can suppose that S100A6 is important for actin filament dynamic rearrangements. Specific isoforms of tropomyosin, e.g. Tpm1.8, can assist S100A6 in this function. We have also demonstrated that S100A6 binds to monomeric G-actin, therefore it is possible that binding of S100A6 to G-actin within the cell compartment containing dynamic actin filaments can enhance depolymerization and shortening of the filament. Because all interactions are Ca^2+^-dependent, S100A6 can be engaged in rebuilding of the actin cytoskeleton in connection with Ca^2+^ signaling pathways. This and the previous data are consistent with the idea that S100A6 regulates the architecture of the actin cytoskeleton, however, more studies are necessary to decipher exact mechanisms of this process.

Our results place S100A6 among the family of actin-binding proteins and extend the list of S100 proteins, which interact with actin cytoskeleton. Another example of an S100 protein interacting with cytoskeletal components is S100A4, which was shown to bind non-muscle myosin heavy chain IIA, tropomyosin and actin, and through these interactions it was proposed to increase cell migration/invasiveness^27^*.* In turn, the S100A8/S100A9 tetramer was found to promote F-actin cross-linking^28,29^. Ca^2+^-dependent interaction of S100A8/S100A9 with components of the cytoskeleton were shown to be involved in migration, degranulation or phagocytosis of activated monocytes and neutrophils. Another member of S100 family, S100A12, a protein constitutively expressed in neutrophils and inducible in macrophages and smooth muscle cells, modulates interaction between cytoskeletal elements and the cell membrane^30,31^*.* Also, S100A2, a protein mainly present in epithelial cells, was proposed to play a direct modulatory role in the organization of the actin cytoskeleton via a Ca^2+^-sensitive interaction with muscle and non-muscle tropomyosins^32^*.* Thus, regulation of the cytoskeleton organization by S100 proteins occurs via interaction with actin and actin binding proteins, and via interaction with tubulin. For instance, it has been reported that S100A8/S100A9 control microtubule reorganization during transendothelial migration of phagocytes^29^ while S100A6 affects microtubules in Wharton's jelly mesenchymal stem cells^33^. Taken together these data show that members of S100 family emerge as important regulators of the cytoskeleton and multiple cellular functions driven by the cytoskeleton dynamics.

In conclusion, our studies show that S100A6 may contribute to the regulation of the organization and functional properties of the microfilaments through direct interactions with actin and tropomyosin.

## Materials and methods

### Cell culture

NIH3T3 mouse fibroblasts were cultured as described by Słomnicki and Leśniak^10^. Cells were grown in Dulbecco’s Modified Eagle’s Medium (DMEM) (Sigma Aldrich) supplemented with 10% fetal bovine serum (FBS) (Gibco), 100 U/ml penicillin, 100 µg/ml streptomycin (Sigma Aldrich) in an incubator at 37ºC and 5% CO_2_. Cells were passaged when confluent and used for experiments 24 h later. For the pull-down assay the protein lysates from NIH3T3 fibroblasts were prepared in buffer containing 20 mM Tris pH 7.5, 50 mM NaCl, 1 mM DTT, 2 mM CaCl_2_, 1 mM MgCl_2_ and protease inhibitors (Roche). Cells were homogenized using a syringe with a needle (30-gauge, 0.5 × 15). Lysates were centrifuged and the supernatant fractions (300 µg of protein estimated using Bradford’s procedure) were applied to the CNBr-Sepharose-S100A6 or CNBr-Sepharose-empty resins.

### Proteins

Purification of the S100A6 protein was performed as described by Słomnicki et al.^34^*.* S100A6 concentration was determined spectrophotometrically at 280 nm using the absorption coefficient 0.439 mg ml^−1^ cm^−1^ at 280 nm and molecular weight 10 000 Da.

Actin was isolated from New Zealand rabbit back and hind leg muscle acetone powder according to Spudich and Watt^35^*.* It was kept in the form of G-actin in a buffer containing 2 mM HEPES, pH 7.5, 0.2 mM ATP, 0.1 mM CaCl_2_, 0.2 mM DTT and 0.02% NaN_3_ (G-buffer). The concentration of G-actin was determined spectrophotometrically by using the absorption coefficient 0.63 mg ml^−1^ cm^−1^ at 290 nm and molecular weight 42 000 Da.

Tropomyosin isoforms (Tpm1.6 and Tpm1.8) were expressed in bacteria BL21 (DE3) cells. Expression of Tpm1.6 and Tpm1.8 was induced with IPTG^36^*.* The cells were harvested and the proteins were purified by ion exchange chromatography as described before^37^*.* Tpm1.6 had an Ala-Ser N-terminal extension, a modification that was previously introduced to increase actin affinity of this isoform^38^. Both tropomyosin isoforms were unacetylated at the N-termini. Concentration of the tropomyosin was determined spectrophotometrically at 280 nm using molar extinction coefficient 17 880 for Tpm1.6 and 8 940 for Tpm1.8. The coefficients were calculated from the amino acid sequences in Expasy (https://web.expasy.org/protparam/).

### Enzyme-linked immunosorbent assay (ELISA)

ELISA assay was performed according to Jurewicz et al.^33^. G-actin, Tpm1.6, Tpm1.8 or BSA (400 ng/well) were coated onto 96-well microtiter plates in 50 μl of Coating Buffer (CB: 100 mM Na_2_HPO_4_, 100 mM NaH_2_PO_4_ pH 8.1). After overnight incubation at 4 °C, the solution was removed and the wells were washed with TBS-T. The remaining adsorption sites were blocked with TBS (10 mM Tris pH 7.5, 10 mM NaCl, 0.05% Tween 20) containing 10% BSA for 3 h at room temperature. Then, increasing amounts of S100A6 were added in 100 μl of Reaction Buffer (RB: 10 mM NaCl, 10 mM Tris pH 7.5, 5% glycerol, 1 mg/ml BSA, 0.2 mM CaCl_2_, 0.2 mM ATP) or (10 mM Tris pH 7.5, 50 mM NaCl, 2 mM MgCl_2_, 5% glycerol, 1 mg/ml BSA, 1 mM CaCl_2_ or 2 mM EGTA), in the actin or tropomyosin-binding assay, respectively. After overnight incubation at 4 °C, the solution was removed. Following the washing procedure with TBS-T, polyclonal anti-S100A6 antibodies, diluted 1:500, (non-commercial) were added and incubation was carried out for 3 h at room temperature. This was followed by addition of a secondary goat anti-rabbit antibody (Sigma Aldrich) diluted 1:7,000. The analysis of bound antibodies was performed by colorimetric detection with TMB peroxidase EIA substrate kit (Bio-Rad). The absorbance at 450 nm was measured using a Bio-Rad microplate reader.

### Cross-linking experiment

Crosslinking with the use of a zero-length cross-linking reagent, EDC, was performed as described by Jurewicz et al.^39^. S100A6 (30 μM) was mixed with G-actin (7.5 μM) in 15 μl of a buffer containing 2 mM HEPES, pH 7.6, 0.5 mM ATP and 0.2 mM CaCl_2_ or 2 mM EGTA. EDC (Sigma Aldrich) was added from fresh stock to 3 mM final concentration and activated by 2 mM NHS. In control reactions, S100A6 and G-actin were cross-linked separately or were incubated without the cross-linker. After 1 h incubation at 25 °C, the reaction was terminated by addition of DTT to a final concentration of 11 mM. Half of the reaction mixture was analyzed by 10% SDS-PAGE stained with Coomassie brilliant blue R250.

### Co-sedimentation assay

Binding of S100A6 to F-actin or the F-actin–tropomyosin complex was studied by co-sedimentation. To remove protein aggregates, S100A6 was ultracentrifuged at 200,000×*g* before the assay and its concentration was measured in the resulting supernatant. Binding of S100A6 to 3 µM F-actin alone or saturated with 0.5 µM Tpm1.6 or Tpm1.8 was analyzed by addition of S100A6 at concentrations from 1–20 µM (corresponding to 0.3–6.7 S100A6:F-actin molar ratio). The assay buffer contained 10 mM Tris, pH, 7.5, 50 mM NaCl and 1 mM CaCl_2_. Protein mixtures were incubated for 15 min at room temperature and then ultracentrifuged for 1 h at 200,000×*g*. The proteins in the pellets and in supernatants were separated on 12% SDS-PAGE. The gels were stained with Coomassie brilliant blue G250. Protein bands were scanned in ChemiDoc MP (Bio-Rad) and their density was quantitated using the EasyDens software (Cortex Nova, Bydgoszcz, Poland). The concentration of unbound S100A6 (free S100A6) remaining in the supernatant was calculated from the standard curve obtained on the basis of the density of S100A6 samples of known concentrations. The saturation curves were plotted as an increase in S100A6:actin densitometric ratios in the function of S100A6:F-actin molar. The binding curves were obtained by fitting the experimental points either to liner regression (S100A6 binding to F-actin alone) or to hyperbolic equation (S100A6 binding to F-actin–tropomyosin complex) in SigmaPlot, version 12.5 (Systat Software Inc., Chicago, IL, USA). In order to compute the apparent binding constant (K_app_) and the Hill cooperativity coefficient (α^H^), the S100A6:actin densitometric ratios were normalized by dividing each S100A6:actin ratio by the maximal value of the ratio at saturation. The thus calculated fractional saturation of the actin filament with S100A6 was plotted as a function of free, unbound S100A6 concentration measured for each sample in the supernatant. The experimental points were fit to the Hill equation (Eq. 1) in SigmaPlot version 12.5 to obtain K_app_ and α^H^.

1 $${\text{v}} = {\text{n}}\left[ {{\text{S1}}00{\text{A6}}} \right]^{{\upalpha {\text{H}}}} \,{\text{K}}_{{{\text{app}}}} \,{}^{{\upalpha {\text{H}}}} {/1} + \left[ {{\text{S1}}00{\text{A6}}} \right]^{{\upalpha {\text{H}}}} \,{\text{K}}_{{{\text{app}}}} \,{}^{{\upalpha {\text{H}}}}$$

where *v* is the fraction of S100A6 bound to actin, *n* is the maximal saturation of the filament with S100A6, and [S100A6] is the concentration of free, unbound S100A6. The average values and standard errors of the binding parameters were from the statistical data reported by SigmaPlot for each curve. Significance of the differences between S100A6 binding to actin in the absence and presence of tropomyosin was analyzed by one way Anova test in SigmaPlot.

### Preparation of CNBr-Sepharose-S100A6 affinity resin and pull-down assay

The coupling of purified recombinant S100A6 protein to CNBr-Sepharose (GE Healthcare) was carried out according to the manufacturer instructions as described by Słomnicki et al.^34^*.* At the same time a control resin with no coupled protein (CNBr-Sepaharose-empty) but blocked with Tris, was prepared.

G-actin or Tpm1.6/Tpm1.8 (purified as described above) were applied to the CNBr-Sepharose-S100A6 affinity resin in a buffer containing 2 mM HEPES, 0.2 mM ATP and 0.2 mM CaCl_2_ or in a buffer containing 10 mM Tris pH 7.5, 30 mM NaCl, 2 mM MgCl_2_, 1 mM CaCl_2_, respectively, and the samples were incubated on a rotating wheel for 1 h at 4 °C. The resins were extensively washed with the same buffer and in the case of tropomyosin additionally with this buffer but containing 150 mM NaCl, 500 mM NaCl and finally with 1 M NaCl. Then, proteins bound in a Ca^2+^-dependent manner were eluted with buffer containing 4 mM EGTA instead of CaCl_2_, precipitated with cold acetone and analyzed by SDS-PAGE. Gels were then stained with Coomassie brilliant blue R250.

In the second experimental approach, protein lysate from NIH3T3 fibroblasts was prepared in buffer containing 20 mM Tris pH 7.5, 50 mM NaCl, 1 mM DTT, 2 mM CaCl_2_, 1 mM MgCl_2_ and protease inhibitors (Roche), and then applied to the resins as described above. After incubation on a rotating wheel for 2 h at 4 °C, the resins were extensively washed with this buffer and then with the same buffer but supplemented with 250 mM NaCl. The proteins bound in a Ca^2+^-dependent manner were eluted with buffer containing 4 mM EGTA instead of CaCl_2_. The elution fractions were precipitated with cold acetone and analyzed by SDS-PAGE and immunoblotting using anti-actin and anti-tropomyosin antibodies (see below).

### SDS-PAGE and immunoblotting

Gel electrophoresis with 10%, 12% or 15% (w/v) polyacrylamide containing 0.1% SDS (SDS-PAGE) was performed by the method of Laemmli^40^*.* Separated proteins were electrotransferred onto nitrocellulose membrane and identified using mouse monoclonal anti-actin-peroxidase (Sigma Aldrich, diluted 1:10,000), mouse monoclonal anti-tropomyosin antibodies, clone TM311 (Sigma, product No. T2780, diluted 1:1,000). After washing with TBS-T buffer (50 mM Tris pH 7.5, 200 mM NaCl, 0.05% Tween 20) the blots were allowed to react with goat anti-mouse IgG (Jackson Immunoresearch Laboratories) secondary antibodies conjugated to horseradish peroxidase, diluted 1:10,000. After three washes with TBS-T and two washes with TBS blots were developed with the ECL chemiluminescence kit (Amersham Biosciences) followed by exposure against an X-ray film.

### Proximity ligation assay (PLA)

For visualization of S100A6–actin and S100A6–tropomyosin complexes, a proximity ligation assay (PLA) (In situ PLA Technology, Olink Bioscience) was applied^33^. NIH3T3 fibroblasts were grown on glass cover slips previously coated with poly-l-lysine (50 μg/ml) for 24 h. The reaction with primary antibodies (rabbit polyclonal anti-S100A6—non-commercial) 1:60 and anti-actin (mouse monoclonal, Sigma Aldrich) 1:400 or anti-tropomyosin (mouse monoclonal, Merck) 1:400 was carried out for 2 h at room temperature. After washing, the incubation with oligonucleotide-conjugated antibodies: anti-rabbit PLA plus and anti-mouse PLA minus probes was conducted for 1 h at 37 °C in a humidity chamber. All following steps were performed according to the manufacturer protocol and with the reagents and media provided in the PLA kit. Immunofluorescence staining was analyzed under a Zeiss Spinning Disc Confocal Microscope (Carl Zeiss GmbH).

## Supplementary information

Supplementary information.

## Abbreviations

K_app_: Apparent binding constant
BSA: Bovine serum albumin
DTT: Dithiothreitol
EDC: 1-Ethyl-3-(3-dimethylaminopropyl)carbodiimide
IPTG: Isopropyl ß-D-1-thiogalactopyranoside
NHS: N-hydroxysuccinimide
PAGE: Polyacrylamide gel electrophoresis
PBS: Phosphate buffer saline
PLA: Proximity ligation assay
SDS: Sodium dodecyl sulfate
Tpm: Tropomyosin
TBS: Tris buffer saline
TBS-T: Tris buffer saline containing Tween 20
