# Supplementary material for: Binding of S100A6 to actin and the actin–tropomyosin complex
Source: Sci Rep. 2020 Jul 30;10:12824. doi: 10.1038/s41598-020-69752-y (PMC7393103; doi:10.1038/s41598-020-69752-y)
Supplement: Supplementary file 1 — Supplementary information. [file 41598_2020_69752_MOESM1_ESM.docx]

Supplementary Information File to the manuscript entitled:

**Binding of S100A6 to actin and the actin - tropomyosin complex**

Ewelina Jurewicz^1^, Katarzyna Robaszkiewicz^2^, Joanna Moraczewska^2🖂^, Anna Filipek^1🖂^

^1^Nencki Institute of Experimental Biology, Polish Academy of Sciences, 3 Pasteur Street, 02-093 Warsaw, Poland

^2^Kazimierz Wielki University, Department of Biological Sciences, 12 Poniatowskiego Street, 85-671 Bydgoszcz, Poland

Corresponding authors:

Anna Filipek

Nencki Institute of Experimental Biology, Polish Academy of Sciences, 3 Pasteur Street,

02-093 Warsaw, Poland; e-mail: [a.filipek@nencki.edu.pl](mailto:a.filipek@nencki.edu.pl)

Joanna Moraczewska

Kazimierz Wielki University, Department of Biological Sciences, 12 Poniatowskiego Street, 85-671 Bydgoszcz, Poland; e-mail: [joanna.moraczewska@ukw.edu.pl](mailto:joanna.moraczewska@ukw.edu.pl)

**Key words:** actin, tropomyosin, S100A6, microfilaments, interaction

**Running title:** Binding of S100A6 to microfilaments


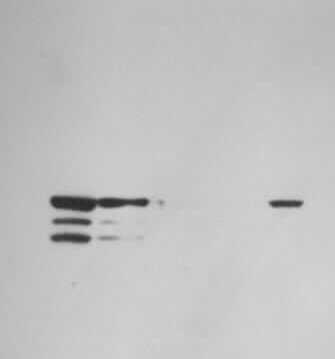


Fig. 1A, upper panel


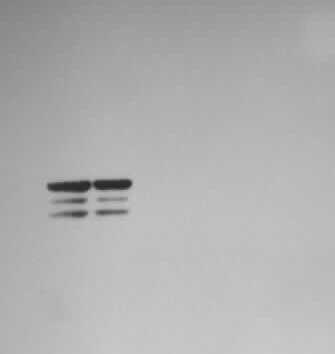


Fig. 1A, lower panel


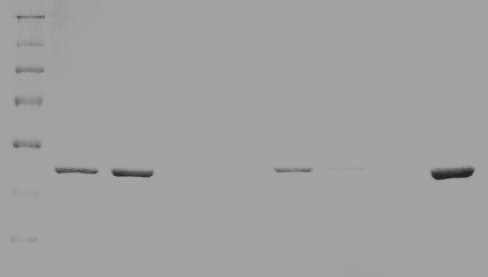


Fig. 2A Left part - lower panel; right part - upper panel.


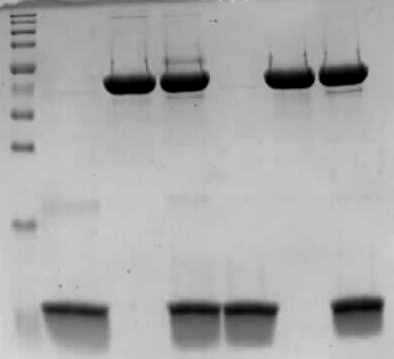


Fig. 2C, left panel


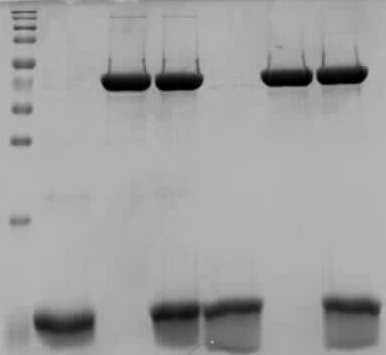


Fig. 2C, right panel


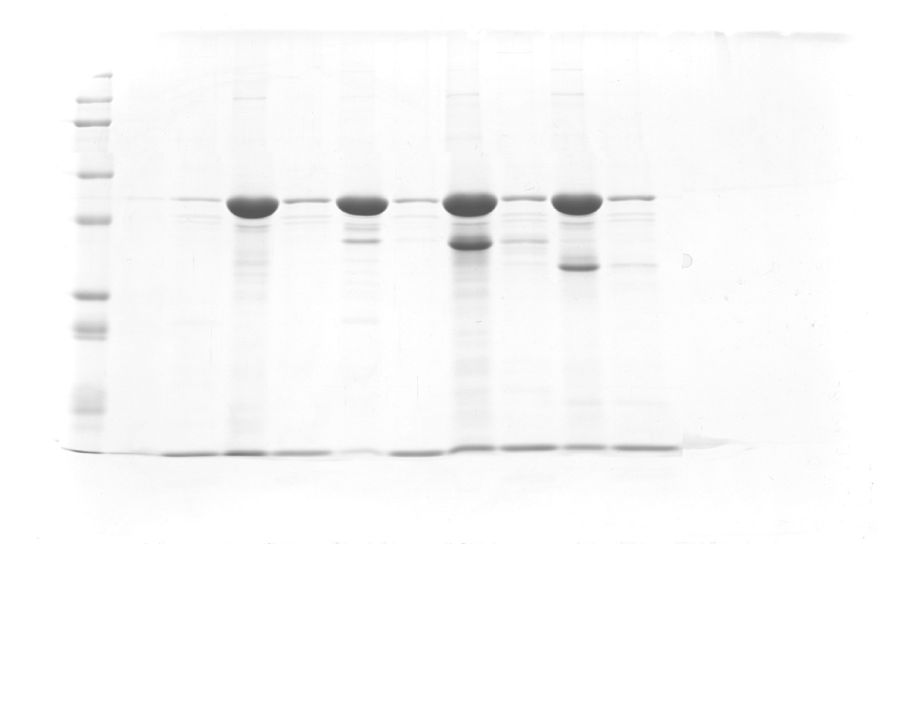


Fig. 3C


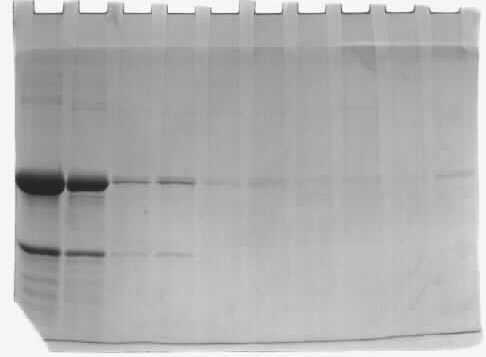


Fig. 4A, 1^st^ panel from the top


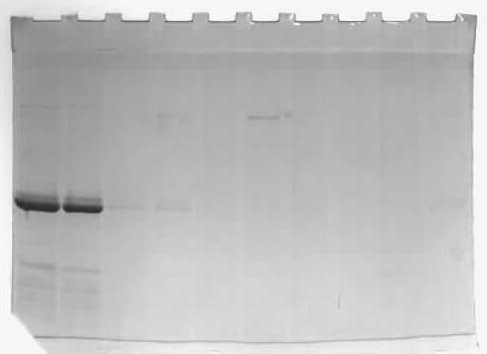


Fig. 4A, 2^nd^ panel form the top


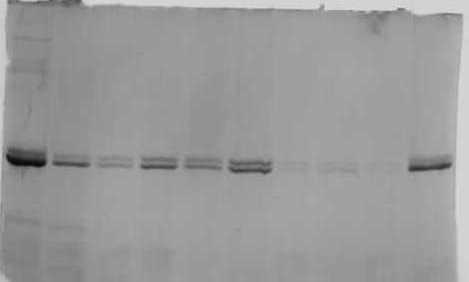


Fig. 4A, 3^rd^ panel from the top


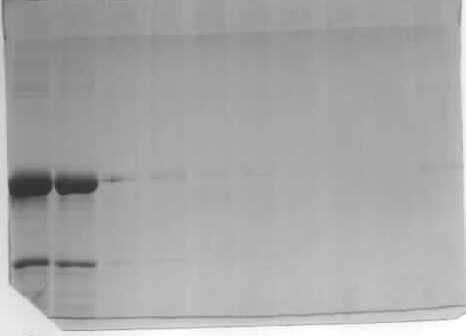


Fig. 4A, 4^th^ panel from the top


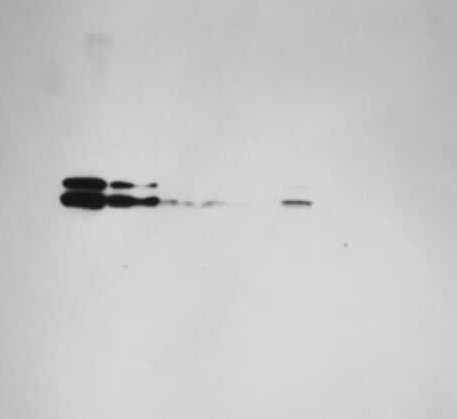


Fig. 5A, upper panel


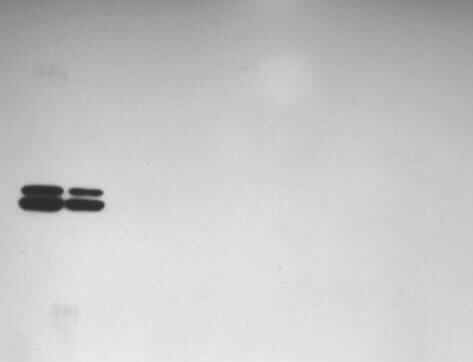


Fig. 5A, lower panel
